# Separation efficiency maximization in acoustofluidic systems: study of the sample launch-position

**DOI:** 10.1039/c8ra08860h

**Published:** 2018-11-20

**Authors:** Valerio Vitali, Tie Yang, Paolo Minzioni

**Affiliations:** University of Pavia, Dept. of Electrical, Computer and Biomedical Engineering Via Ferrata 5A 27100 Pavia Italy paolo.minzioni@unipv.it +39 0382 422 583 +39 0381 985 221; School of Physical Science and Technology, Southwest University Chongqing 400715 China

## Abstract

The development of lab-on-chip microfluidic systems based on acoustic actuation, and in particular on the acoustophoretic force, has recently attracted significant attention from the scientific community thanks, in part, to the possibility of sample sorting on the basis of both geometrical and mechanical properties. It is commonly recognized that sample prefocusing and launch-position optimization have a substantial effect on the performance of these systems but a clear explanation of how these two parameters influence the system efficiency is still missing. In this manuscript we discuss the impact of both the sample launch position and the sample distribution at the input by the theoretical analysis of a simplified system and by numerical simulations of realistic configurations. The results show that the system performance can be greatly improved by selecting the proper microchannel dimensions and sample-launch position, offering relevant guidelines for the design of micro-acoustofluidic lab-on-chip devices.

## Abstract 2

We theoretically and numerically show how to optimize the separation-efficiency of acoustofluidic systems by a non-trivial selection of sample-injection position.

## Graphical abstract

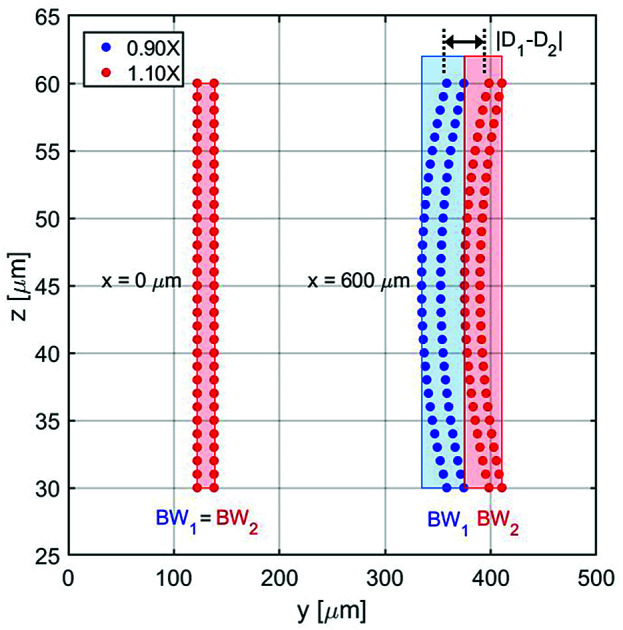

## 1 Introduction

Over the past twenty years, microfluidic and lab-on-chip techniques have generated significant research interest and successfully permeated into many different fields, with particular attention being paid to lab-on-chip systems for cytology applications.^1^ Their inherent micro-size characteristic provides a natural environment to detect, and manipulate cells, even for analysis at a single cell level.^2^ Multiple functionalities have already been integrated within a single chip, thanks to the inclusion of different sensors and sample-actuation mechanisms, such as those based on dielectrophoresis,^3,4^ optical forces,^5–7^ cavitation bubbles^8,9^ and magnetic forces.^10,11^ An actuation system which is currently attracting considerable attention is that based on the interaction between the sample and acoustic waves. On-chip acoustofluidics, which combines the use of ultrasonic acoustic waves with the advantages of microfluidic systems, has become an extremely active field and several review papers,^12,13^ tutorials^14^ and books^15^ have been dedicated to this field.

Acoustofluidics has been successfully applied to many different research studies, ranging from micro-droplet production and manipulation to micro-particle (or cell) sorting, focusing, separation, mixing and arraying.^16–27^ Among these possible applications, cell separation has attracted a lot of attention including substantial effort devoted towards enabling the isolation of circulating tumor cells from human blood samples.^28–36^

Several groups have demonstrated the possibility of isolating target cells from a given sample containing a mix of cells with different characteristics using acoustofluidics.^37,38^ A theoretical analysis of particle separation efficiency in acoustophoretic devices was recently reported and it showed that both intrinsic factors, related to the sample itself, and extrinsic factors, related to the microfluidic system, can strongly affect the separation result. Among all the factors, the sample launch position into the active region (*i.e.* where the acoustophoretic force is present) plays a critical role.^39^

The aim of this paper is to investigate how the launch position of the sample inside the channel changes the separation efficiency, and to demonstrate how the best launch position depends on different parameters: the aspect ratio of the channel, the cross-section occupied by the sample distribution at the channel input and the radius of the target bead/cell sample. In the following we focus our attention on investigating the challenging situation where the micro-objects to be selected and separated show a small deviation of their properties from the other beads/cells flowing along the channel.

## 2 Background knowledge

This section reports some of the basic information about system geometry along with the fundamental equations that determine the performance of the configurations considered in the paper.

### 2.1 Description of analyzed configuration

**Fig. 1:**
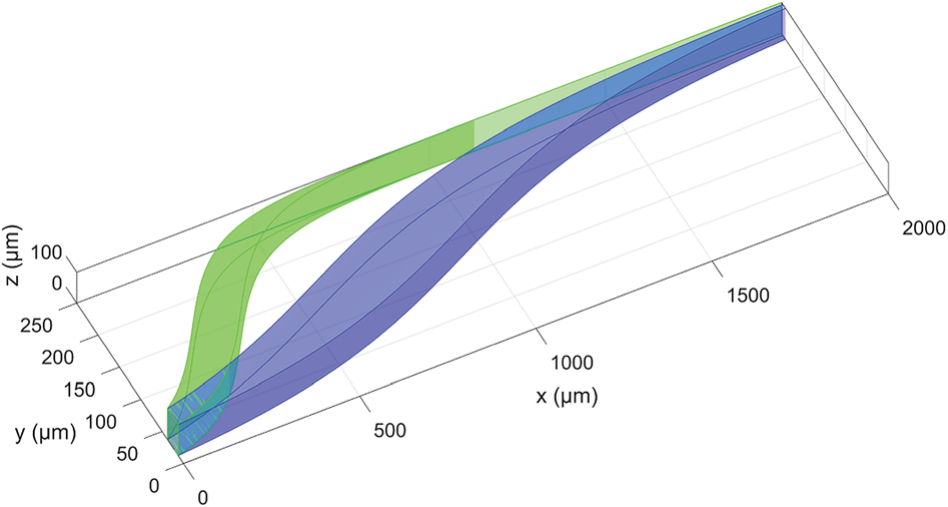
3D scheme of the particle separation mechanism considered in this study. The blue and the green volumes represent the positions occupied by beads with different acoustic contrast factors (80% and 100% of that associated with polystyrene beads in water) while they flow along the *x*-direction. It should be noted that this figure is only used to introduce the analyzed configuration and the reference system. The *z*-dependence of the fluid velocity profile has not been taken into account in the calculation of the shown trajectories.

We take into consideration the simple situation of a rectangular microchannel, with a cross-sectional area of 0.09 mm^2^ and with variable aspect ratio (defined as the ratio between width and height, *w*/*h*), where an acoustic standing-wave (with a single pressure node and resonating along the microchannel width^40^) is introduced. We consider a sample-injection area different from the main inlet of the buffer fluid, positioned in the “right-half” of the microchannel, as illustrated graphically in Fig. 1, and we analyze the sample movement across the channel (towards the channel's center) due to the acoustic radiation force caused by the scattering of the acoustic wave by the particles. In real-world situations the sample-injection area yields a certain statistical distribution of the sample starting position (*y*_0_,*z*_0_); we thus want to analyze the impact of the initial position uncertainty (Δ*y*_0_,Δ*z*_0_) on the achievable separation between two sample populations with slightly different properties.

It is worth underlining that in our analysis we neglect the effect of gravity, which can be helpfully used to produce a sample separation in the vertical direction,^41^ and the acoustic streaming-induced drag force (generally relevant for particles much smaller than cells).

### 2.2 Fundamental equations and parameter definitions

The reference system used throughout the paper is shown in Fig. 1: the fluid flows in the *x* direction, the acoustic wave resonates along the *y*-direction (corresponding to the channel width) and the microchannel height is in the *z*-direction.

One of the main parameters affecting the movement of particles exposed to acoustic waves is the so called acoustic contrast factor (*φ*), which is given by the following equation where *ρ*_p_ and *ρ*_f_ are the densities of the suspended microparticles and fluid, respectively, and *β*_p_ and *β*_f_ are the corresponding compressibilities.^40^1
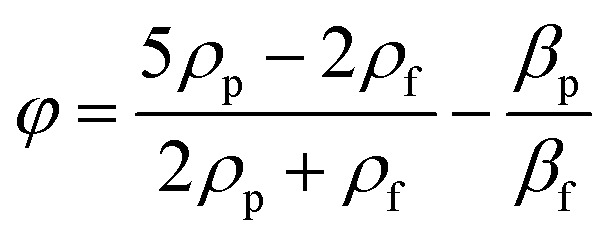


The acoustic contrast factor is one of the main quantities appearing in the expression of the acoustophoretic force applied to a microsphere in a plane standing wave, see eqn (2), where *R* is the beads/particles radius, *y* is its position in the transverse direction, *E*_ac_ is the acoustic energy density in the microchannel, and *k*_*y*_ represents the acoustic wave number.2
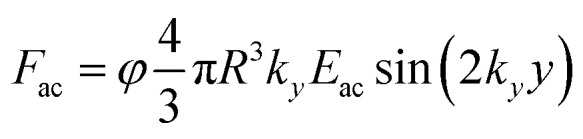


By imposing the Stokes drag force to be equal to the acoustic force it is possible to calculate the transverse coordinate of the particle as a function of time, as shown in eqn (3).^40^ In that equation we identify as *y*_0_ the position, along the *y*-axis, occupied by the particle when it enters the area of the microchannel where the acoustic wave is present.3
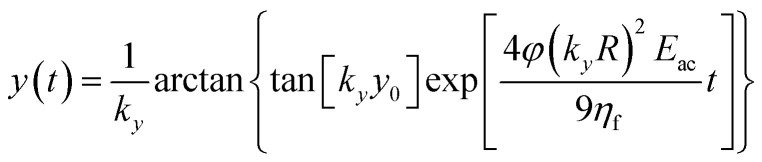


Regarding the particle movement in the *x*-direction, we assume that it moves together with the fluid, whose velocity *v*_*x*_(*y*,*z*) in the microchannel is given by eqn (5).^39^ To derive that equation we assume a rectangular microchannel of height *h*, width *w*, we identify with ∇*P* the pressure gradient and with *v*_c,∞_ a constant factor, defined by eqn (4).4
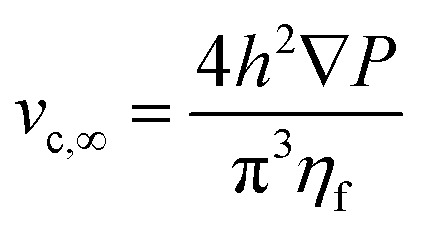
5
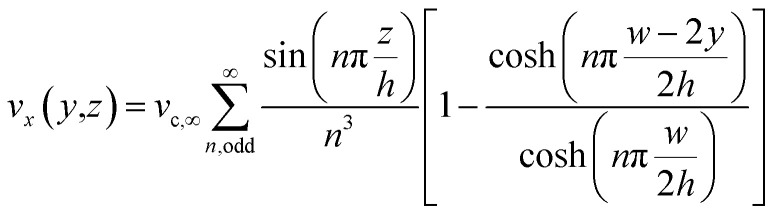


Regarding the *z*-direction, conversely, we assume that no significant movement occurs during the time that the microbeads flow along the microchannel. We also initially set Δ*z*_0_ = 0, even if we will remove this assumption in Section 4.2.

The above equations allow us to calculate the movement in 3D of a particle flowing in the considered microfluidic system, and thus they also allow us to define the bandwidth (BW), displacement (*D*) and separation-efficiency (SE) parameters, as already reported in ref. 39. To briefly recall those definitions, we identify as *D*_*i*_(*x*) the distance traveled by beads belonging to the *i*-th population along the *y*-direction with respect to the starting position, *y*_0_, and we name BW_*i*_(*x*) the spreading, in the *y*-direction, of the *i*-th population.

**Fig. 2:**
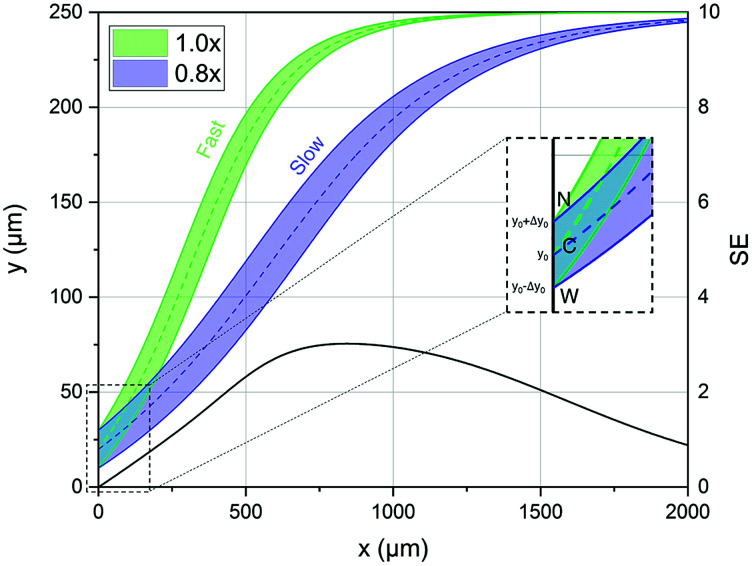
Left scale: transverse position along the width direction of two bead populations with different acoustic contrast factors (80% and 100% of that associated with polystyrene beads in water) while they flow along the *x*-direction. The inset shows a zoomed-in view of the injection region (“C” stands for the center of the injection area, “W” stands for the border of the injection area closer to the microchannel wall and “N” stands for the border closer to the node of the acoustic wave). Right scale: the SE calculated according to eqn (6), and as a function of the *x* coordinate, shows a peak at about 800 μm where the two populations are well separated.

Starting from these two quantities, the SE parameter is calculated, at each position in the *x* direction, by the ratio of two distances as shown by eqn (6). As a numerator we use the difference between the distances traveled by two beads, with different characteristics, and injected in the microchannel at the same position *y*_0_. Conversely at the denominator we sum the two single-side bandwidths with the bead radius. As a result the SE parameter calculated by eqn (6) is greater than 1 when the two populations are completely separated by a distance larger than one bead radius (see Fig. 2).6
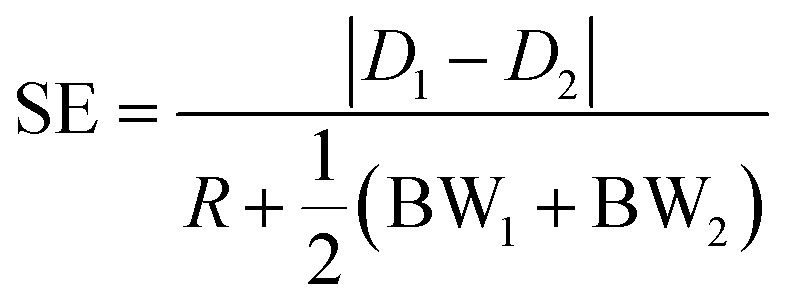


An analysis of the intrinsic (*i.e.* sample-related) and extrinsic (system-related) parameters affecting the SE can be found in ref. 39.

## 3 Theoretical analysis: uniform flow-speed

We start our analysis by noticing from eqn (5) that every time the distance from the border is larger than twice the channel's height (*i.e. y* > 2*h*) the ratio between the two hyperbolic cosine functions in eqn (5) becomes negligible. As a consequence, in this condition the fluid speed through the channel cross-section does not depend on the *y*-coordinate. When a large aspect-ratio microchannel (*i.e.* having *h* ≪ *w*) is considered, the area where the flow speed depends on the *y*-coordinate (*y* < 2*h*) represents a negligible portion of the cross-section and it is thus possible to assume that the beads movement along the *x*-direction does not depend on the position along the *y*-direction. As a consequence, the sample separation depends on eqn (3) and we can thus consider such a configuration as a 1D-system. To simplify the writing of eqn (3), but without losing important dependences, we can introduce the following definitions:7
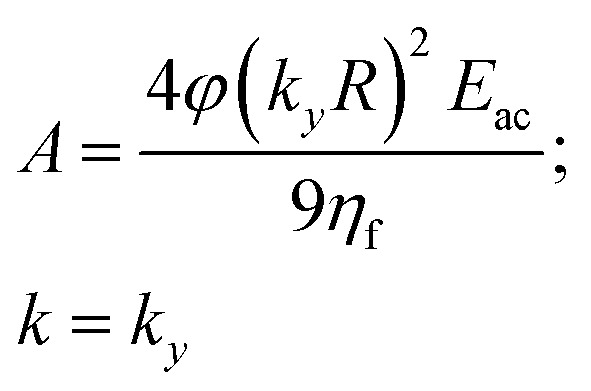


This allows us to rewrite eqn (3) as:

8 *y*(*t*) = *k*^−1^ arctan{tan[*ky*_0_]exp[*At*]}

We use this compact form to develop an analytical study of the simplified 1D-system which, although an over-simplification, provides useful insights into the impact of different parameters on the separation efficiency (SE), as defined in eqn (6). Using this notation, a change in the particle properties is reflected by a change of the *A* parameter, and it is thus possible to define an average *A* and a deviation Δ*A*. Similarly, as anticipated in Section 2, Δ*y*_0_ represents the uncertainty of the input position, *i.e.* the maximum variation from the desired value *y*_0_.

### 3.1 Performance analysis of 1D-systems

In order to derive the SE dependence on the *A* and *y*_0_ parameters we consider two different populations (a “fast” one and a “slow” one; F/S) and for each of them we consider three different “injection points”: the center of the injection area (C), the border of the injection area closer to the microchannel wall (W) and the border closer to the node of the acoustic wave (N). We can thus write 6 equations similar to eqn (8), where we modify *A* in *A* ± Δ*A* to include the effect of the “fast” and “slow” population, and we use *y*_0_, *y*_0_ − Δ*y*_0_ and *y*_0_ + Δ*y*_0_ to take into account the C, W and N starting positions respectively (see inset of Fig. 2):9
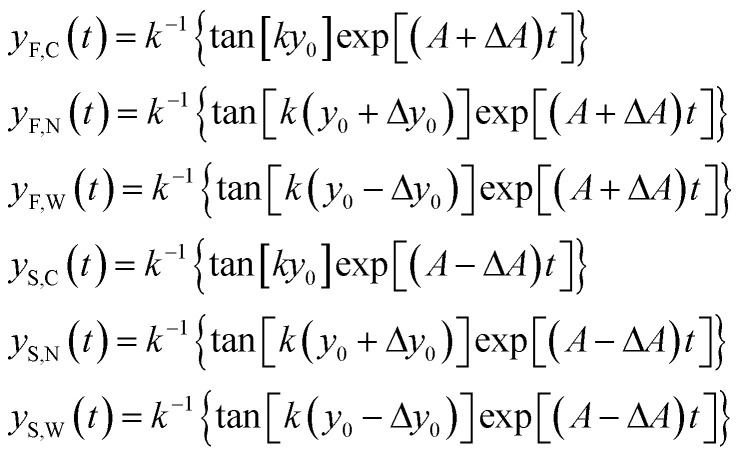


Using this definition, it is possible to rewrite eqn (6) as:10
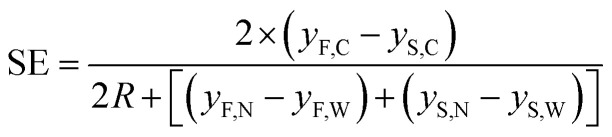


If we then approximate the differences appearing in the eqn (10) with the corresponding first-order differential terms we obtain:11
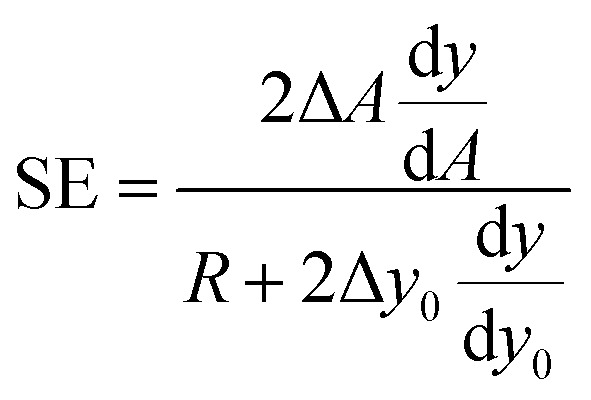


The above equation can be rewritten in a more useful form by calculating the two derivatives so as to obtain an explicit expression for the SE evolution as a function of time, or of flown-distance, as we consider uniform flow speed in the channel. If we then set Δ*A* = Δ*A*_r_ × *A*, where Δ*A*_r_ is the relative variation of the *A* parameter we obtain:12
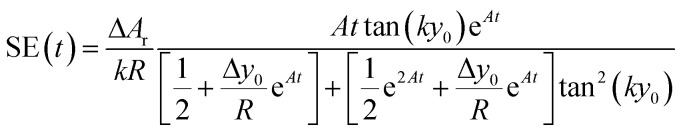


In the following we identify with an asterisk (*) the values yielding the best performance, thus 
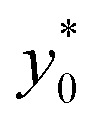
 and t* are the launch-position and time-instant corresponding to the maximum separation efficiency (SE*) achievable between two populations. In particular, thanks to eqn (12), it is possible to derive some non-trivial considerations about 
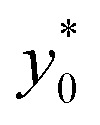
, t* and SE*:

• C.(1) The role of Δ*A*_r_:

With the given hypothesis the SE(*t*) (and hence SE*) is directly proportional to Δ*A*_r_, but Δ*A*_r_ has no effect on the *y*_0_ value maximizing the SE. It thus means that the optimal launch position of the sample in the channel 
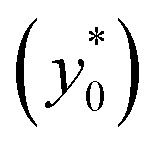
 doesn't depend on how large the sample property variations are.

• C.(2) The maximum-separation instant (t*):

The time instant giving the maximum SE cannot be explicitly calculated as the resulting equation is transcendental, but we observe that in eqn (12) the time always appears in the product *At*. This implies that the maximum-separation instant t* is inversely proportional to *A*, *i.e.* the product At* does not depend on *A*. It is thus useful to rewrite eqn (12) in a more compact way by defining the following terms:13
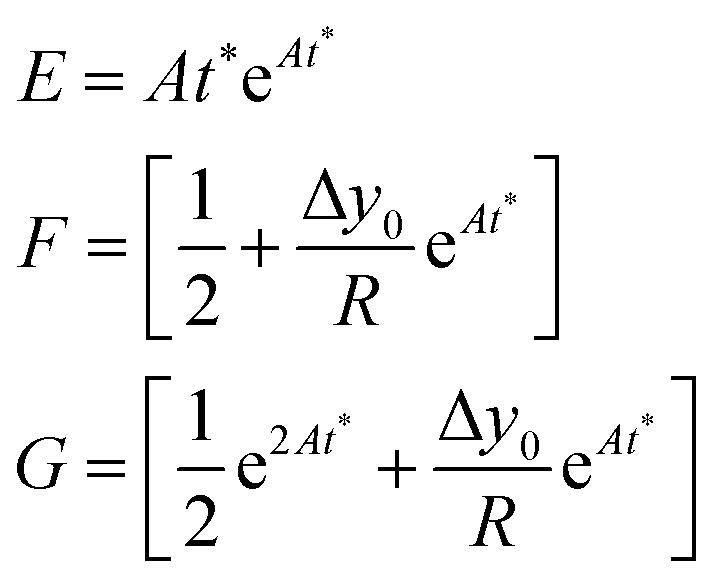


The SE achieved at t* can thus be calculated as shown in eqn (14), which has two important characteristics: *E* is a constant while both *F* and *G* only depend on the value of Δ*y*_0_/*R*.14
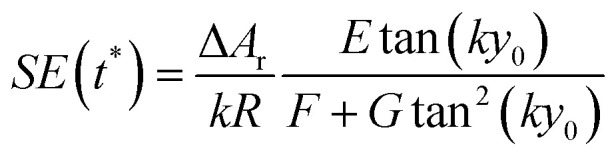


• C.(3) The ratio Δ*y*_0_/*R*:

Following the above considerations we observe that if both Δ*y*_0_ and *R* are multiplied by the same quantity, SE (t*) is left unmodified and thus also the optimal launch position 
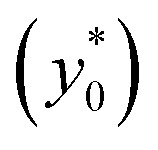
 doesn't change. Nevertheless, it is important to highlight that even if we keep Δ*y*_0_/*R* constant, a variation of *R* affects both the value of t* and that of SE(t*) as they are proportional to *R*^−2^ and *R*^−1^.

**Fig. 3:**
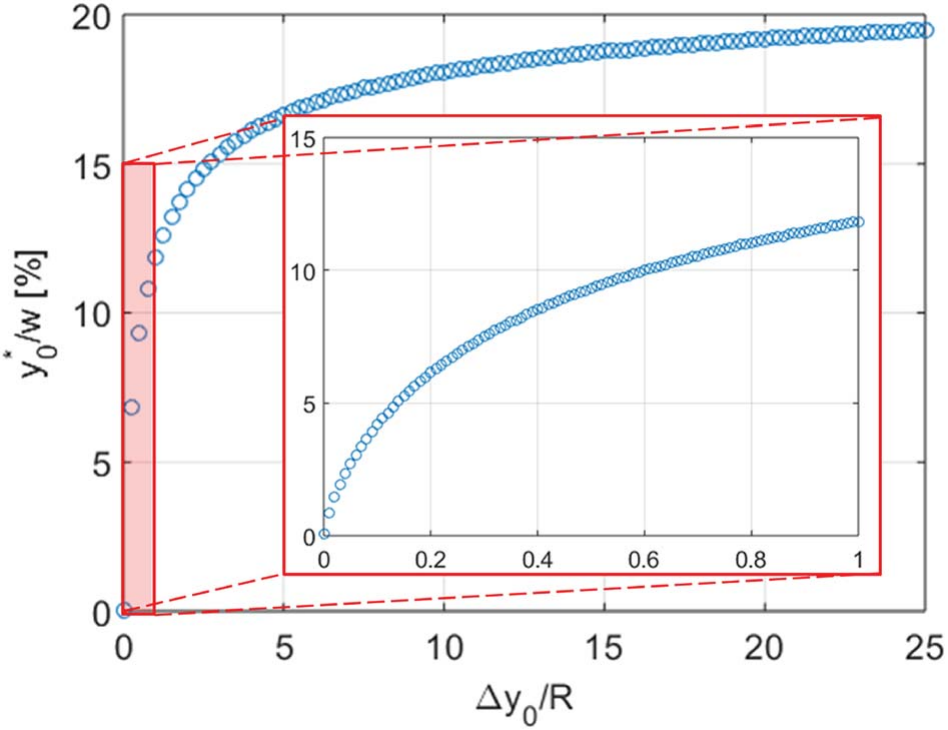
Best sample-injection position 
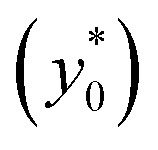
, expressed as a percentage of the total channel width *w*, as a function of Δ*y*_0_/*R*. The inset shows an expanded view of the low-Δ*y*_0_/*R* region (Δ*y*_0_/*R* ≤ 1). 
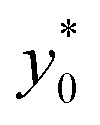
 increases rapidly in the initial part and then almost saturates around 20% of *w* when Δ*y*_0_/*R* ≥ 5.

• C.(4) The 
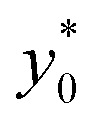
 value:

In this simplified situation 
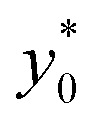
 depends only on the ratio Δ*y*_0_/*R*. By analyzing Fig.(3) it is interesting to notice that the ideal injection position is close to the border when the ratio Δ*y*_0_/*R* is almost zero, it rapidly increases when Δ*y*_0_/*R* grows from 0 to 1 (going from 0% to 12% of *w*) and then it almost saturates around 20% of *w* when Δ*y*_0_/*R* gets much larger than 1.

After this study of the 1D system it becomes relevant to analyze, by means of numerical simulations, how the system properties are affected by moving to a 2D and 3D system, so as to verify if the above considerations are still valid.

## 4 Numerical simulations: results and discussion

The comparison with a more realistic situation, by means of numerical simulations, is carried out in a two-step process. As a first step (Section 4.1) we introduce the presence of a non-uniform flow-speed profile across the channel width (*y*-direction using the reference system shown in Fig. 1) and we compare the results by varying the aspect-ratio (*w*/*h*) of the microchannel cross-section. In this case the analysis of the results obtained at large aspect ratio can also be used to assess the validity of the results obtained analytically in the previous section. In this “2D-case” we neglect the dependence of the flow speed (*v*_*x*_) on the *z*-direction which is equivalent to assuming that the sample is injected at the half-height of the microchannel and neglecting the vertical sample dispersion. Subsequently, as the second step (Section 4.2), we also include in our model the dependence of *v*_*x*_ on the vertical direction *v*_*x*_(*y*,*z*) and the vertical-dispersion of the sample at the microchannel input (Δ*z*_0_), so as to mimic a realistic situation. This allows us to investigate the impact on the achievable SE of the vertical position and spreading of the sample inlet, allowing us to derive some interesting design rules. In both cases we carry out the simulations using typical parameter values corresponding to a water-suspension of polystyrene (PS) microbeads. The parameter values are reported in Table 1.

**Table 1:** Main simulation parameters

	Symbol	Value
**Water**		
Density	*ρ*_f_	998 kg m^−3^
Compressibility	*β*_f_	4.48 10^−10^ Pa^−1^
Viscosity	*η*_f_	8.94 10^−4^ Pa s
Sound speed	*ν*_f_	1483 m s^−1^

**Beads**		
Density	*ρ*_p_	1050 kg m^−3^
Compressibility	*β*_p_	2.49 10^−10^ Pa^−1^
Beads radius	*R*_p_	3.75 10^−6^ m

**Microchannel**		
Pressure gradient	∇*P*	20 Pa m^−1^
Acoustic energy density	*E*_ac_	1.0 J m^−3^

All the simulations were carried out using a custom MATLAB script based on the equations reported in Section 2.2. The beads trajectory at the x–y (2D systems) and x–y–z (3D systems) coordinates as a function of time were calculated combining eqn (3) and (5), thanks to the use of “ODE45” function of MATLAB. The trajectories were calculated over a time vector, composed by 2000 uniformly spaced values, whose time-step depends on microbeads properties and on the channel geometry, as they impact on the time required by beads to reach the microchannel center. As a general indication, the calculation of each point appearing in the figures from 4 to 6 required about half an hour of computation time on a 4-cores processor at 3.50 GHz and with 16 GB of RAM.

It is possible to investigate different microchannel aspect-ratios in two different ways: keeping one dimension fixed and varying the other one (*e.g.* fixed height and variable width), or by simultaneously modifying both dimensions so as to keep the cross-sectional area constant. In the following, unless otherwise specified (in the final part of Section 4.2), we keep the microchannel area fixed at 9 × 10^−2^ mm^2^, corresponding to a square microchannel with a side of 300 μm. The dependencies and trends observed for this specific area are of general validity.

### 4.1 2D-systems approximation

The first phase of these simulations always involved the identification of the proper time-interval to be considered and of the required spatial- and temporal-resolution. For each parameter-set of the microchannel-beads system, we considered different launch positions (*y*_0_) and for each of them we derived the corresponding SE, thus allowing us to identify the optimal launch position 
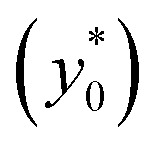
.

**Fig. 4:**
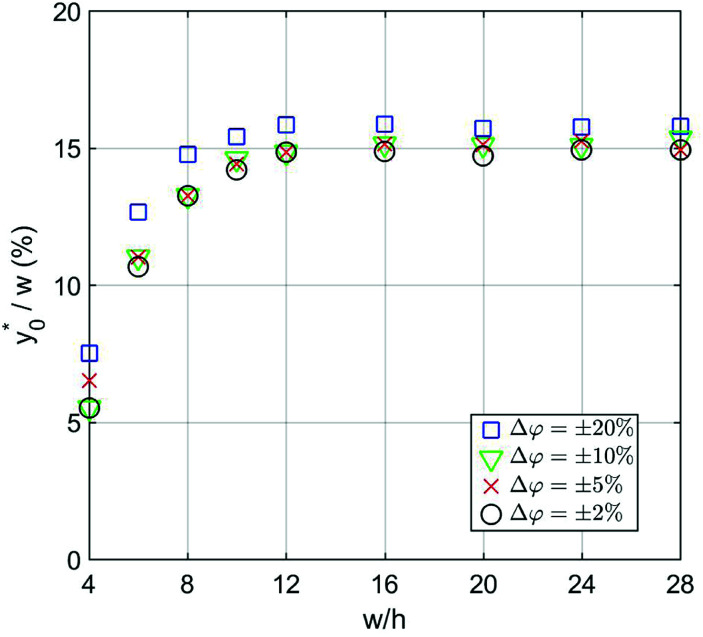
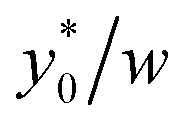
 (Best injection position expressed as a percentage of the channel width) as a function of channel's aspect ratio. Parameters used for this simulation: *R* = 3.75 μm; *φ* = 0.5; Δ*y*_0_ = 10 μm. Values in the legend correspond to Δ*A*_r_ value in eqn (12).

The numerical analysis was initially carried out to verify whether or not the considerations expressed in Section 3 are still valid in the 2D case. In order to verify the previously reported consideration C.(1), we considered in our numerical simulations distinct beads populations having a *φ* (see eqn (1)) different from that of standard PS. In Fig. 4 we show the 
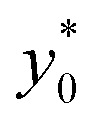
 values as a function of the microchannel aspect-ratio (*w*/*h*) and considering four different bead-population pairs.

The first pair is composed by beads having an acoustic contrast factor (*φ*) equal to 98% and 102% of the nominal PS value, and it is thus indicated as ±2% in the legend (corresponding to the Δ*A*_r_ value in Section 3). In an analogous way, we considered population-pairs with an increased difference of *φ*, up to the ±20% case, which corresponds to bead-populations having an acoustic contrast factor equal to 80% and 120% respectively of that of PS. As reported in Section 3, it is possible to notice that the 
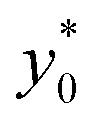
 position doesn't depend on how large the sample properties variations are, provided that the difference is not too big. As it is evident considering the ±20% case, if the *φ* variation becomes too large the 
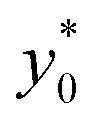
 position may start to vary, as the first-order approximation used to derive eqn (11) is not sufficient anymore.

Nevertheless, as the most critical separation situation is when small differences are present between the sample populations, this limitation is not particularly relevant for our study. It is also interesting to notice that the 
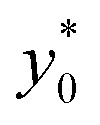
 value depends on the aspect ratio of the microchannel cross-section (indicated as *w*/*h* in the figures), but it becomes almost constant when *w* > 10*h*, as it approaches the 1D-situation theoretically analyzed in Section 3. As a comparison it is interesting to notice that data used to create Fig. 4 yield a ratio Δ*y*_0_/*R* ≈ 2.7 which corresponds in Fig. 3 to 
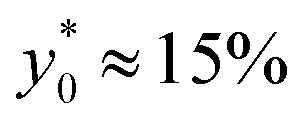
, exactly matching the numerically calculated position for large aspect-ratios.

**Fig. 5:**
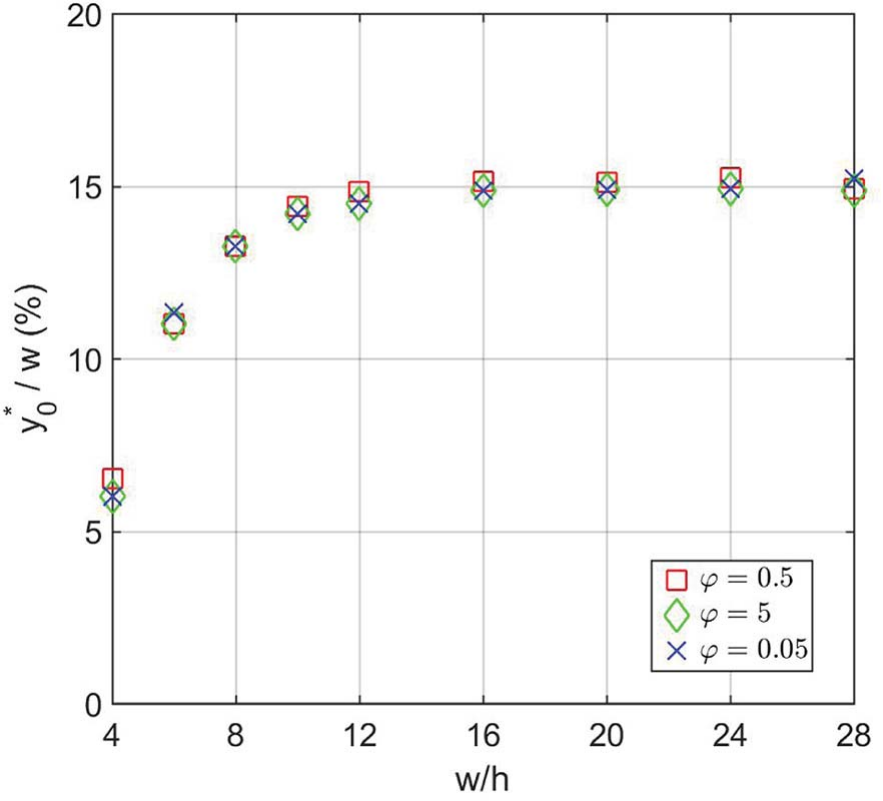
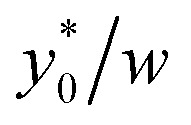
 *vs.* channel's aspect ratio (*w*/*h*) when three different values of *φ* are considered: 0.5, 5 and 0.05. Other simulation parameters: *R* = 3.75 μm; Δ*φ* = ±5%; Δ*y*_0_ = 10 μm.

Subsequently we also verified that the 
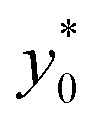
 value does not depend on the absolute value of *φ*. To analyze this aspect, we considered three different bead-population pairs, with largely different values of the nominal *φ* factor (0.05, 0.5 and 5), while keeping Δ*φ* = ±5%. The results show that no change of the 
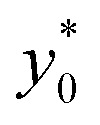
 value is induced by modifying the nominal *φ* of the populations, independently of the channel aspect ratio (see Fig. 5).

We then moved to verify the dependence of 
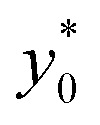
 on the Δ*y*_0_ and *R* parameters (*i.e.* consideration C.(3) of Section 3). According to what previously reported we expect the 
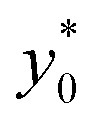
 value to depend on Δ*y*_0_/*R*, but not on Δ*y*_0_ and *R* separately. To assess this dependence, we compared the ideal 
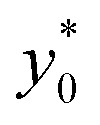
 for five different configurations of Δ*y*_0_ and *R*, while keeping constant the acoustic contrast factor of the two populations (*φ* = 0.5; Δ*φ* = ±5%). The five parameter-set considered in the numerical simulations are schematically reported in Table 2. We included three different combinations of Δ*y*_0_ and *R* yielding the same Δ*y*_0_/*R* ratio (8/3), and two different combinations yielding a four-times increase and decrease of the Δ*y*_0_/*R* value (32/3 and 2/3 respectively).

**Table 2:** Simulation parameters used to assess the dependence of 
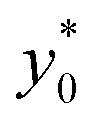
 on Δ*y*_0_ and *R*

Color	Symbol	Δ*y*_0_ [10^−6^ m]	*R* [10^−6^ m]	Δ*y*_0_/*R*
Blue	Triangle	10	3.75	2.67
Red	Diamond	20	7.5	2.67
Green	Square	5	1.875	2.67
Black	Circle	20	1.875	10.67
Cyan	Triangle	5	7.5	0.67

**Fig. 6:**
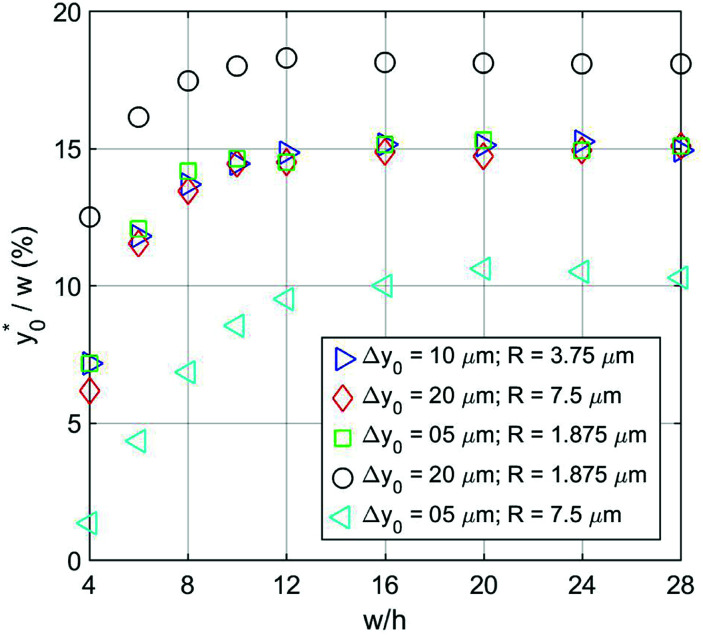
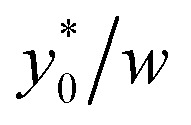
 as a function of *w*/*h* considering different Δ*y*_0_/*R* combinations. The values of Δ*y*_0_ and *R* are as reported in Table 2. Other simulation parameters: *φ* = 0.5; Δ*φ* = ±5%.

The results clearly highlight that even in this case, as in the 1D situation previously considered, the optimal launch position depends on Δ*y*_0_/*R*, and is thus unmodified if both values are multiplied by the same factor (see Fig. 6).

**Fig. 7:**
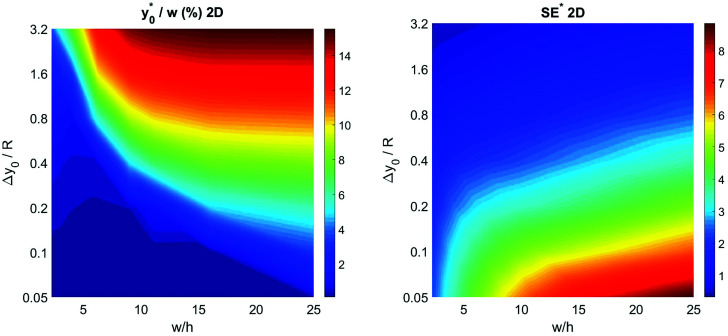
Optimal injection position (
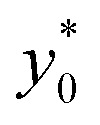
, left) and corresponding separation efficiency (SE*, right) as a function of Δ*y*_0_/*R* and of channel aspect ratio in the 2D case.

To complete the 2D-approximation analysis of the launch position we created a figure to show the overall dependence of the 
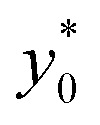
 parameter on Δ*y*_0_/*R* and *w*/*h*, which are the only two parameters affecting the 
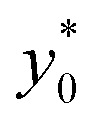
 value. The result of the numerical simulations is reported in Fig. 7 (left panel) as a color-map. Calculations were carried out considering a nominal *φ* = 0.5, Δ*φ* = ±5% and *R* = 5 μm, but the reported results have a much more general validity as derived by the above reported analysis.

In the same conditions we also calculated the SE* value, defined according to eqn (6), achievable by proper selection of the injection point, as a function of Δ*y*_0_/*R* and *w*/*h*. The obtained results, reported in right panel of Fig. 7, show the benefit of using large aspect ratios and the advantages given by a reduction of Δ*y*_0_, which can be achieved, as an example, by using a prefocusing section.^42^

The results of this analysis demonstrate two important aspects: (i) the importance of optimizing the launch position 
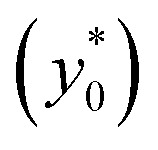
 to improve the SE and (ii) that the 
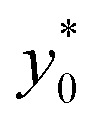
 value, expressed as a percentage of the microchannel width, depends only on microchannel aspect ratio (*w*/*h*) and on the Δ*y*_0_/*R* ratio, but not on other sample factors. Anyway, it is important to highlight that the whole analysis reported up to this section completely neglects the vertical dimension of the microchannel.

### 4.2 Extension to 3D-systems

**Fig. 8:**
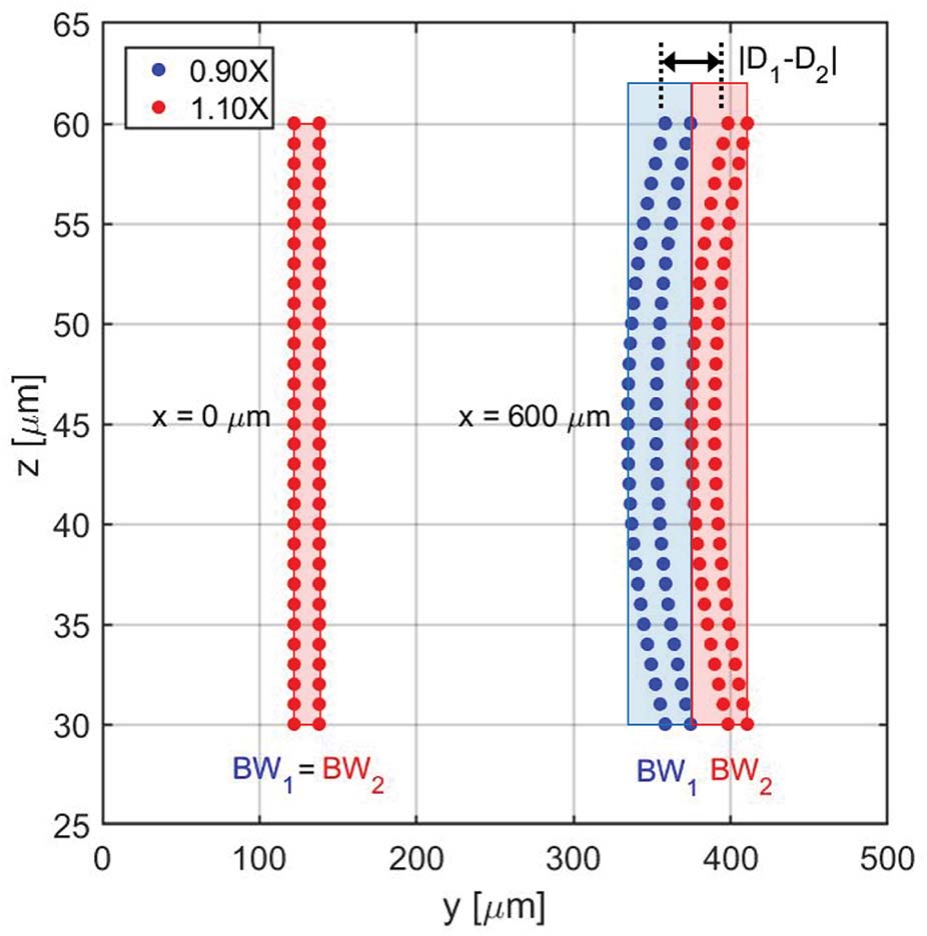
Schematic illustration of micro-particles acoustic separation in the 3D case. Parameters: *w* = 1000 μm; *h* = 90 μm; water medium; compressibility and density of polystyrene (*φ* = 0.5); Δ*φ* = ±10%; *R* = 5 μm; Δ*y*_0_ = 8 μm; Δ*h* = ±15 μm.

In this section we analyze a realistic 3D-system, including also the role played by the microchannel vertical dimension, and we thus add in our model two important effects: the dependence of flow speed on the *z*-coordinate and the fact that the sample distribution has a non-zero dimension also along the vertical direction. As a first step, we need to properly re-define the SE parameter: as beads flowing at different heights have different velocities, even if we assume to have a rectangular distribution of beads at the input, the positions occupied while flowing along the microchannel produce a curved distribution of beads at any other section, as it can be seen in Fig. 8. Since we are interested in having a realistic evaluation of the system performance, we assume the cross-section of the “sample-extraction” port to have a rectangular shape, independently of the curved areas corresponding to the beads positions. We thus keep the definition of the SE unmodified, as in eqn (6), and we include the effect of the curved beads-distribution by re-defining the BW values and |*D*_1_ − *D*_2_| (see Fig. 8).

In particular we define the BW parameter of each population as the maximum distance (in the *y*-direction) between two beads, *i.e.* considering at each section along the *x*-axis the bead closer to the microchannel wall (at half of the channel height) and the one closer to the microchannel center (and closer to microchannel floor). The distance |*D*_1_ − *D*_2_|, which represents the distance between the “centers” of the two beads distributions, is calculated as the distance between the centers of the two population-bands.

It is important to notice that, as it is evident by Fig. 8, choosing an injection height different from the center of the channel can only worsen the system performance. The flow-speed gradient in fact becomes larger as we move away from the middle-height position and thus the beads distribution becomes wider. For this reason, we considered in our analysis the impact of a vertical spreading Δ*y*_0_ while keeping the center of the injection channel fixed at half-height of the microchannel. Regarding the Δ*y*_0_ parameter it should be noticed that it is possible to define it as a given percentage of the microchannel height, or by its own value (in μm).

To keep consistency with the previous analysis we initially consider the case of Δ*y*_0_ defined as a fixed percentage of the microchannel height. We decided to start our analysis considering a Δ*z*_0_ equal to 5% of the channel height, while keeping all the other parameters set as for the final 2D simulations: nominal *φ* = 0.5, Δ*φ* = ±5% and *R* = 5 μm. As in the previous case we calculated the optimal launch position 
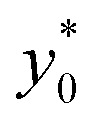
, and the corresponding separation efficiency (SE*) as a function of the microchannel aspect ratio and of the Δ*y*_0_/*R* value.

**Fig. 9:**
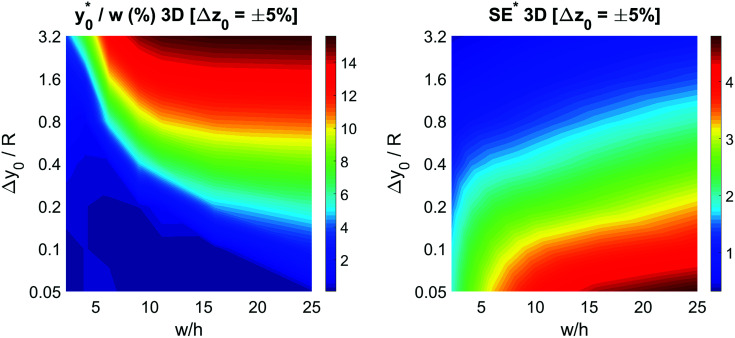
Left: consider the sample having a certain vertical distribution around the half-height of the channel. The vertical spread is equal to ±5% of the channel height. Optimal injection position 
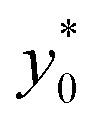
 as a function of the ratio Δ*y*_0_/*R* and of the channel aspect ratio in the 3D case. Right: corresponding maximum value of SE* as a function of the ratio Δ*y*_0_/*R* and of the channel aspect ratio in the 3D case.

The data reported in Fig. 9 (left panel and right panel) show two partially surprising results: the 
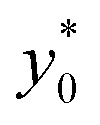
 obtained in the 3D case exactly matches that obtained in the 2D approximation and also the SE* figure matches that obtained in the 2D case, once rescaled by a constant factor. The reason for these results is that the presence of a vertical spreading implies the presence of beads flowing at a different height, where the flow speed is simply scaled (by a factor smaller than 1) with respect to the flow speed at half-height of the channel. As no distortion of the speed profile is introduced, the 
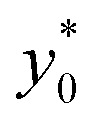
 value for beads flowing at half height and for those flowing at any distance from the channel bottom is the same, provided that beads' interaction with the bottom surface can be neglected. A direct consequence of this is that even analysis carried out using a larger vertical spreading (*e.g.* Δ*z*_0_ equal to 10% or 15%) would yield the same results and thus do not bring any additional information.

The obtained values show that even a minor vertical spreading can have a significant impact on the achievable SE in case of microchannels with high aspect ratio: as an example, a 5% vertical spreading in the microchannel with aspect ratio 25 corresponds to a Δ*z*_0_ as small as ±3 μm, and yields a SE* reduction almost by a factor of 2. On the other side, the use of microchannels with a smaller aspect ratio, although yielding a lower SE* value in the ideal case of Δ*z*_0_ = 0, is expected to be significantly more tolerant to the vertical spreading. We thus investigated the performance of microchannels with different aspect-ratios while fixing Δ*z*_0_ equal to ±5 μm and ±10 μm.

**Fig. 10:**
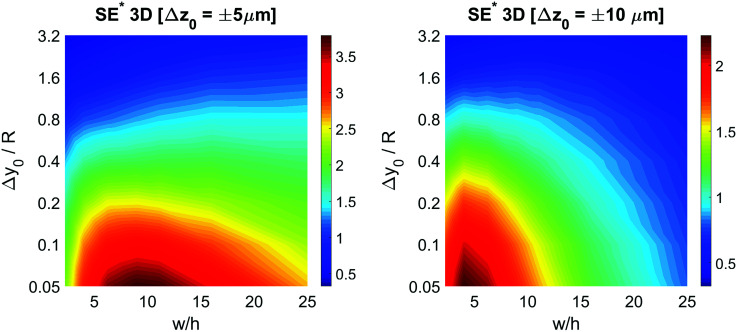
Left: consider the sample having a certain vertical distribution around the half-height of the channel. The vertical spread is equal to ±5% μm. SE* as a function of the ratio Δ*y*_0_/*R* and of the channel aspect ratio in the 3D case. Right: consider the sample having a certain vertical distribution around the half-height of the channel. The vertical spread is equal to ±10 μm. SE* as a function of the ratio Δ*y*_0_/*R* and of the channel aspect ratio in the 3D case.

The 
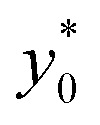
 color-maps obtained in these conditions do not add any relevant information with respect to Fig. 9 and they are thus not reported in the manuscript. Conversely, it is interesting to analyze the data reported in Fig. 10, showing the achievable SE in the above described conditions and with a fixed value of Δ*z*_0_. The reported maps highlight that, once Δ*y*_0_/*R* and Δ*z*_0_ are given, it is possible to identify the ideal channel cross-section and then the achievable SE.

It is interesting to notice that while in the 2D case an aspect ratio as large as possible was desirable (see right panel of Fig. 7), in the 3D case the presence of a non-negligible Δ*z*_0_ suggests the use of higher channels, so as to mitigate the effect of the vertical spreading of the sample. As a consequence of the necessity to find a trade-off between the mitigation of horizontal and vertical spreading, the ideal aspect ratio has a non-obvious dependence on both Δ*y*_0_/*R* and Δ*z*_0_.

Additionally, it is worth mentioning that in the above reported discussion we exclusively focused our attention on the acoustic radiation force, applied on the flowing particles because of the sound-waves scattering, while we neglected the acoustic streaming effect and the related drag-force. Following the analysis reported by Muller *et al.*^43^ it is possible to show that, in order to neglect the acoustic streaming effect, the particle diameter must exceed by a few times the boundary-layer thickness *δ*.15
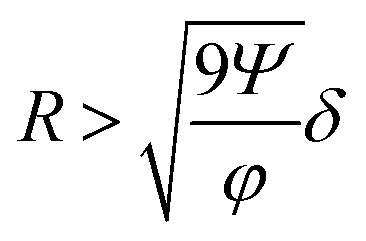
where *Ψ* is a parameter related to the microchannel geometry and that in case of planar walls is equal to 3/8. With some simple passages, this condition can be written as a limitation on the microchannel width (*w*) expressed by the below reported equation, where *η* represents the dynamic viscosity of the fluid, *ρ* is the fluid density and *c*_0_ is the sound-wave speed in the fluid:16
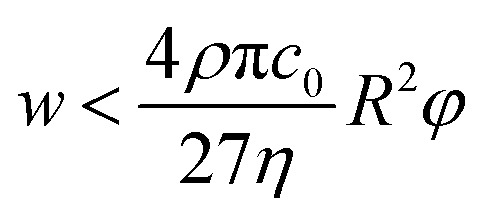


As a reference value, considering cells with *φ* = 0.15 and *R* = 3 μm the maximum value of *w* allowing to neglect the acoustic streaming effect is ≈1000 μm, which may thus impose a limitation on the achievable microchannel aspect-ratio (*w*/*h*).

## 5 Conclusions

In this work we described the results of an analytical and numerical investigation regarding the acoustic separation of microbeads. In particular, we focused on the study and optimization of the sample-launch position in order to maximize the separation efficiency in the challenging situation where the micro-objects to be separated are characterized by a small deviation of their properties from the other micro-particles flowing along the channel. We showed that the best sample injection position depends on different factors (the aspect ratio of the channel, the cross section occupied by the beads distribution at the sample inlet and the radius of the bead). The optimization method presented in this study allowed us to derive important design rules which can be applied to free-flow microfluidic separation systems independently on the presence or absence of pre-focusing stages and strategies.

It is interesting to notice that thanks to a careful optimization of the injection position of the sample, high SE values can be obtained even in case of no prefocusing techniques. This allows largely simplifying the design and the operation of the microfluidic systems.

## Conflicts of interest

There are no conflicts of interest to declare.

## Supplementary Material
